# No Clear Differences between Organic or Conventional Pig Farms in the Genetic Diversity or Virulence of *Campylobacter coli* Isolates

**DOI:** 10.3389/fmicb.2017.01016

**Published:** 2017-06-26

**Authors:** Martine Denis, Bérengère Nagard, Valérie Rose, Kévin Bourgoin, Mélina Cutimbo, Annaëlle Kerouanton

**Affiliations:** ANSES, Hygiene and Quality of Poultry and Pig Products Unit, Bretagne-Loire UniversityPloufragan, France

**Keywords:** *Campylobacter*, pig, organic, PFGE, MLST, virulence

## Abstract

To evaluate the impact of pig farm management on the genetic diversity and on the virulence of *Campylobacter coli*, we characterized isolates from 19 organic pig farms (62 isolates) and from 24 conventional pig farms (58 isolates). The 120 *C. coli* isolates were typed using pulsed field gel electrophoresis (PFGE) and multilocus sequence typing (MLST) and the presence of nine virulence genes was screened using real-time PCR. The capacity of adhesion and invasion of 61 isolates (32 from organic and 29 from conventional farms) were then tested on human intestinal *Caco-2* cells. A total of 59 PFGE types and of 50 sequence types (STs) were identified. Twelve PFGE types and nine STs, accounting for 34 and 41.6% of the isolates, respectively, were common between the two production systems with ST854 dominating (18.3% of the isolates). Twenty-nine PFGE types and 25 STs were only found in isolates from organic farms, and 18 PFGE types and 16 STs from conventional farms. No significant differences were found in diversity despite the differences in rearing systems, except at the locus level for the *glnA, gltA*, and *uncA* genes. All isolates, regardless of their origin, carried the *ceuE, iam, ciaB*, and *flaA* genes and more than 95% of the isolates carried the *cadF* and *cdtABC* genes. No significant differences were found in pathogenicity between the two farming systems. The pathogenicity of the *C. coli* isolates was low compared to *C. jejuni* control strains tested. The plasmid gene *virb11* was detected in only 13 isolates from organic farms; these isolates showed greater invasion capacity than those without this gene. Our study indicates that pig farm management does not significantly affect the diversity and the virulence of *Campylobacter coli* isolated from pigs. The common genotypes between conventional and organic farms may indicate that some genotypes are adapted to pigs.

## Introduction

New consumer trends focus on products derived from systems that promote good animal welfare conditions and a high safety level. Organic pig production differs in many ways from conventional pig production, particularly in terms of antibiotic use, herd structure, feeding regimes, access to outdoor areas, and space allowance per pig. More specifically, the European Union regulations for organic farming (Council Directives 2007/834/EC and 2008/889/EC) require that animals have access to an outdoor area. In addition, the preventive use of chemically-synthesized allopathic veterinary medicinal products is not authorized, and may be used only for treatment of sick animals when necessary. If more than one treatment, derived products are no longer considered organic.

Recently, we investigated the carriage of antibiotic-resistant *Escherichia coli* in colons at the slaughterhouse and in feces on organic and conventional pig farms in four European countries (SafeOrganic project, Österberg et al., 2016). We also studied the carriage of resistant *Campylobacter* from the same samples in two European countries (SafeOrganic project, Kempf et al., 2017). In France, the level of antibiotic resistance in *E. coli* and *Campylobacter coli* is lower for organic pig production than for conventional production, suggesting that practices such as little or no use of antibiotics on organic pig farms can affect the level of bacterial resistance. Several studies (Saini et al., 2013; Garcia-Migura et al., 2014) indicate that extensive use of antibiotics produces a selection pressure favoring resistance among commensal bacteria from animals.

Because the management of conventional and organic pig farms has an impact on *Campylobacter* resistance to antibiotics (Kempf et al., 2017), we assumed that the management of these two types of pig production systems (in terms of antibiotic use and access to the outdoors) may also have an impact on the diversity of *Campylobacter* isolates excreted by pigs and on the virulence of these isolates. The more frequent use of antibiotics and confinement of pigs in a building in conventional farming may reduce the number of *Campylobacter* genotypes. In contrast, in organic farming, little or no use of antibiotics and access to an outdoor area may promote the presence of a higher number of *Campylobacter* genotypes. Access to an outside area increases exposure of animals to environmental sources of different microorganisms including *Campylobacter jejuni* (Greig et al., 2015).

Thus in this study, the *C. coli* previously isolated from pigs from organic and conventional farms to test their resistance to antibiotics (Kempf et al., 2017) were typed using two molecular typing methods, and tested for their virulence.

## Materials and methods

### Origin of the isolates

The *Campylobacter* isolates considered in this study were isolated by our laboratory, which is also the French National Reference Laboratory for *Campylobacter* as part of the SafeOrganic project. Sampling and isolation methods for *Campylobacter* are described in Kempf et al. (2017). Briefly, colon contents were sampled at one slaughterhouse from 114 pigs. These pigs came from 31 organic pig batches (56 pigs) and 31 conventional pig batches (58 pigs). These batches involved 21 organic farms and 29 conventional farms, all located within 200 km of the slaughterhouse. Out of the 50 sampled farms, 43 farms were positive for *Campylobacter*: 19 organic farms and 24 conventional farms.

The isolates were kept at −80°C in peptone glycerol broth. They were all identified as *C. coli* and tested for their antibiotic resistance (Kempf et al., 2017). Here, we randomly selected two to three isolates per positive farm for a total of 120 isolates: 62 isolates from 19 organic farms and 58 isolates from 24 conventional farms.

### DNA extraction

The 120 isolates were cultured on blood agar plates (Oxoid, Dardilly, France) for 48 h at 37°C in a micro-aerobic atmosphere (5% O_2_, 10% CO_2_, 85% N_2_). A few colonies from the bacterial culture were used for DNA extraction using the InstaGene® Matrix (BioRad Laboratories, Marnes-la-Coquette France) according to the manufacturer's recommendations. DNA was adjusted to 10 ng/μl and intended for use in PCRs for virulence gene detection, and multilocus sequence typing (MLST) as described below. The remaining colonies were used for genotyping by pulsed-field gel electrophoresis (PFGE).

### Pulsed-field gel electrophoresis (PFGE) and analysis of electrophoretic profiles

DNA preparation, restriction endonuclease digestion with the *Kpn*I enzyme and PFGE were carried out as described by the Campynet protocol (Rivoal et al., 2005). One *Kpn*I restriction profile was obtained for each isolate.

Electrophoretic patterns were compared using BioNumerics v. 6.5 software (Applied Maths, Sint-Martens-Latem, Belgium). Similarities between profiles, based on band positions, were determined by calculating the Dice correlation coefficient, with a maximum position tolerance of 1%. A dendrogram based on the *Kpn*I restriction profiles was constructed to represent the similarities between the isolates in the matrix. Isolates were clustered by the unweighted pair-group method using the arithmetic mean (UPGMA) (Struelens, 1996). Isolates displaying high levels of similarity were clustered together using a threshold of 80% (Denis et al., 2008) and considered as the same PFGE type.

The Simpson's index (D) was determined as described by Hunter (1990), and was given with a 95% confidence interval, as described by Grundmann et al. (2001). This index was used to assess the genetic diversity of the *Campylobacter* populations.

### Multilocus sequence typing (MLST)

The seven housekeeping genes for MLST (*aspA, glnA, gltA, glyA, pgm, tkt*, and *uncA*) were amplified and sequenced according to previously developed experimental conditions (Dingle et al., 2001; Miller et al., 2005). PCR products were cleaned up using the ExoSAP-IT treatment (GE Healthcare), and sequence extension reactions were carried out in BigDye Ready reaction mix according to the manufacturer's instructions. Unincorporated dye terminators were removed using an ethanol precipitation method before the products were analyzed on an ABI Prism 3130 sequencer (Applied Biosystems). The sequences were assembled using the assembler implemented in BioNumerics v. 6.5 software. All allelic sequences were queried against the *C. jejuni* MLST database (http://pubmlst.org/campylobacter). Alleles already present in the database were assigned the numbers given there; novel alleles and sequence types (STs) were submitted to the MLST database and assigned new numbers. STs were assigned into genetically related clusters called clonal complexes (CCs), based on the sharing of four or more alleles with the central genotype that had been identified in previous studies using the BURST algorithm and UPGMA cluster analysis (Dingle et al., 2002).

### Detection of virulence genes

Table 1 shows the nine virulence genes screened in the present study. Eight of the genes are localized on the bacterial chromosome and one on the plasmid (*virB11*). These genes are involved in adhesion/invasion of epithelial cells (*flaA, ciaB, cadF, iam, virB11)*, in the acquisition of iron (*ceuE)*, and in the production of the cytholethal distenting toxin (CDT) (*cdtA, cdtB, cdtC)*.

**Table 1:** Primer sequences for the detection of the nine virulence genes in *Campylobacter coli*.

**Gene**	**Primer**	**Sequence (5′ →3′)**	**Size (bp)**	**References**
*flaA*	flaA2-F	GCTTCAGGGATGGCGATAGCAGAT	533	Moore et al., 2002
	flaA1-R	TTGATCTCTTCAGCCAAAGCTCCAAGT		
*cdtA*	cdtA-cF	TGTCCCACCTGTAATCACTCC	245	This study
	cdtA-cR	CTCTTGCATCTCCAAAAGGTCT		
*cdtB*	cdtB-cF	GAGTGGATGTAGGAGCAAATCG	332	This study
	cdtB-cR	CGTAGAAGAAGGCGGAACAAC		
*cdtC*	cdtC-cF	AGCTTGGATGAATTAGCAGACT	403	This study
	cdtC-cR	TGGCGATACTAGAGTCAGGAAA		
*cadF*	F2B^*^	CTTTGAAGGTAATTTAGATATG	401	Konkel et al., 1999
	R1B^*^	AACTAATACCTAAAGTTGAAAC		
*virB11*	virB11-235	TGTGAGTTGCCTTACCC	240	Zheng et al., 2006
	rev-virB11-F^**^	GCTAGTTTTTCCACTTCCTG		Bang et al., 2004;
*ceuE*	COL3	AATTGAAAATTGCTCCAACTATG	462	Gonzalez et al., 1997
	MDCOL2	TGATTTTATTATTTGTAGCAGCG		Denis et al., 1999
*iam*	Car-F	GCGCAAAATATTATCACCC	519	Carvalho et al., 2001
	Car-R	TTCACGACTACTATGCGG		
*ciaB*	ciaB-cF	GAAAGAAGCTATGGTGTTTTGGT	284	This study
	ciaB-cR	GGATGACCTACTTGYAATGGAGA		

* *Primer modified from the initial primer reported in the Reference*.

** *Reverse sequence of the initial primer (virB11) published by Bang et al. (2004)*.

The presence of these nine virulence genes in the 120 isolates was checked using real-time PCR developed for this study using primers published by Gonzalez et al. (1997), Denis et al. (1999), Konkel et al. (1999), Carvalho et al. (2001), Moore et al. (2002), Bang et al. (2004), or by Zheng et al. (2006) or using primers designed by pour laboratory for this study (Table 1).

Some published primers were slightly modified (one or two bases added or removed) to obtain primers with the same (or very similar) melting temperature (indicated by ^*^ in Table 1). We used also the reverse sequence of the initial primer (*virB11*) published by Bang et al. (2004).

We designed eight primers from a sequence alignment using Multalin v. 5.4.1 (INRA, France) for the detection of the three *cdt* genes (GenBank accession numbers: AB562905, AB274801, AB274800, AB274799, AB274798, AB274797, AB274796, AB274795, AB274794, AB274793, AB182109) and the *ciaB* gene (GenBank accession numbers: HG326877, CP006702, AB433217, CP004066).

The size of the PCR products was estimated by *in silico* PCR (FastPCR online v. 2.07, PrimerDigital) and confirmed after electrophoresis on an agarose gel.

Each PCR was carried out in a total volume of 25 μl with the mix SYBR® Green JumpStart™ Taq ReadyMix™ from Sigma-Aldrich, in which 1 μl of each primer at 10 μM was added. All nine amplifications were done using the same PCR conditions: 35 cycles, each cycle with a first step at 95°C for 1 min, a second step at 56°C for 1 min and a final step at 72°C for 1 min 40 s. The PCRs finished with an incremental step from 60 to 95°C, increasing by 0.5°C every 5 s to obtain the fusion curve.

Three isolates of human origin were used as positive controls (Table 2): *C*. *jejuni* 81–176 (with pVir plasmid) and *C. jejuni* NCTC11168 (without the pVir plasmid) (purchased from the Pasteur Institute Collection, Paris) and *C. coli* 04FM842 (purchased from the French National Reference Center) genetically close to *C. coli* from pigs by PFGE and with all the virulence genes except *virB11*.

**Table 2:** Presence (+) of the nine genes in the three strains used as positive PCR controls.

**Strain**	**Species**	***flaA***	***cdtA***	***cdtB***	***cdtC***	***cadF***	***virB11***	***ceuE***	***iam***	***ciaB***
NCTC 11168	*C. jejuni*	+	−	−	−	+	−	−	−	−
81−176	*C. jejuni*	+	−	−	−	+	+	−	−	−
04FM842	*C. coli*	+	+	+	+	+	−	+	+	+

### Adhesion and invasion assay on human intestinal epithelial cells

For this assay, we selected 61 isolates (32 from the 19 organic farms and 29 from the 24 conventional farms) on the basis of their PFGE profile and ST to ensure good representativeness of the isolates from each farm.

Capacity of adhesion and invasion of the isolates was tested *in vitro* on *Caco-2* human intestinal epithelial cells following the protocol developed in our laboratory by Guyard-Nicodème et al. (2013). *Caco-2* cells (ECACC 86010202) were obtained from the European Collection of Cell Culture (ECACC, Salisbury, UK). Capacity of adhesion and invasion of the isolates was expressed as the percentage of adhesives cells (p_adh) and of invasive cells (p_inv), respectively. For each isolates, the results were the mean of at least two separate determinations.

*C. jejuni* and *C. coli* strains isolated from humans and poultry were also tested to compare the invasiveness of our isolates with other *Campylobacter*. The three human strains were *C. jejuni* 81–176 (with pVir plasmid), *C. jejuni* NCTC11168 (without pVir plasmid), and *C. coli* 04FM842 (without pVir plasmid). The poultry strains were Plouf12 (*C. jejuni* from poultry) and Plouf13 (*C. coli* from poultry), both previously tested on pig in a previous study (Leblanc Maridor et al., 2011), 17MD18, 47MD12 (two *C. coli* from poultry), 54MD16, and 27MD13 (two *C. jejuni* from poultry) isolated from a previous study (Denis et al., 2008), CRL204-08 (*C. coli* from poultry) purchased from the European Reference Laboratory, Uppsala, Sweden. We added also a *C. coli* reference strain isolated from a pig (CIP70.80T) purchased from the collection of Pasteur Institute, Paris, France.

### Data analysis

The distribution of the PFGE types or STs on organic farms was compared to that of conventional farms using the chi-square test of independence in R software (version 3.2.5). For each typing method, we considered the number of isolates from one production system that shared types common with the other system, and the number of isolates found in only one of the two production systems. The distribution was considered statistically different between the two production systems when *p*-values were lower than 0.05.

Using the “comparing partitions” method (http://www.comparingpartitions.info), we compared the distribution of the PFGE types of the 120 isolates with the distribution of the STs of these isolates. We also compared the distribution of the antibiotic resistance (ATB) profiles of the 120 isolates with the distribution of the PFGE types or STs of these isolates. A *p*-value was calculated using the jackknife pseudo-values method. We considered that the PFGE/ST, ATB/PFGE, or ATB/ST associations were weak if their distributions were significantly different (*p* < 0.05).

Results of adhesion to or invasion of *Caco-2* cells were analyzed using the Mann-Whitney test in R software version 3.2.5. A *p*-value lower than 0.05 was considered significant. *C. coli* were also classified into three classes of pathogenicity (low, intermediate and high) using hierarchical clustering with the method “hclust ward D2” implemented in R.

## Results

### Genetic diversity

**Figure 1:**
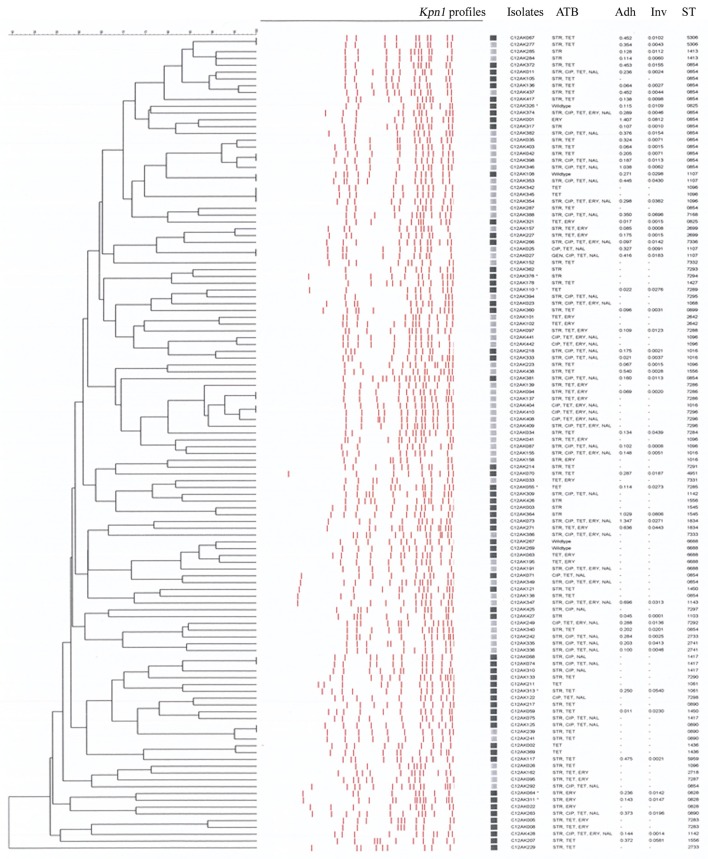
Dendrogram of the *Kpn*1 profiles of the 120 *Campylobacter coli* isolates from organic and conventional pig farms (optimization, 1%; tolerance, 1%; active zones, [7.0–87.0%]). Black squares, isolates from organic pig farm; Gray squares, isolates from conventional pig farms; ATB, profile of antibiotic resistance; Adh, percentage of adhesion on *Caco-2* cells; Inv, percentage of invasion on *Caco*-*2* cells; ST, sequence type.

We observed high genetic diversity for our 120 *C. coli* whatever the typing method. A total of 110 *Kpn*1 PFGE profiles were associated with 59 PFGE types (Figure 1) when clustered at 80% of similarity. The 62 isolates from organic farms showed 41 PFGE types and the 58 isolates from conventional farms showed 30 PFGE types. Organic farms and conventional farms shared 12 PFGE types (12/59) representing 34.16% of the isolates (41/120). Among the 59 PFGE types, 29 were found only from organic farm isolates (40 isolates) and 18 from conventional farm isolates (39 isolates). The diversity of the *Campylobacter* populations was slightly higher in organic isolates with an index of diversity *D* = 0.98 _*CI*95%_[0.97–0.99] than in conventional isolates with *D* = 0.96 _*CI*95%_[0.94–0.98]. The distribution of the PFGE types in the two production systems was not significantly different (χ^2^, *p* = 0.345).

**Figure 2:**
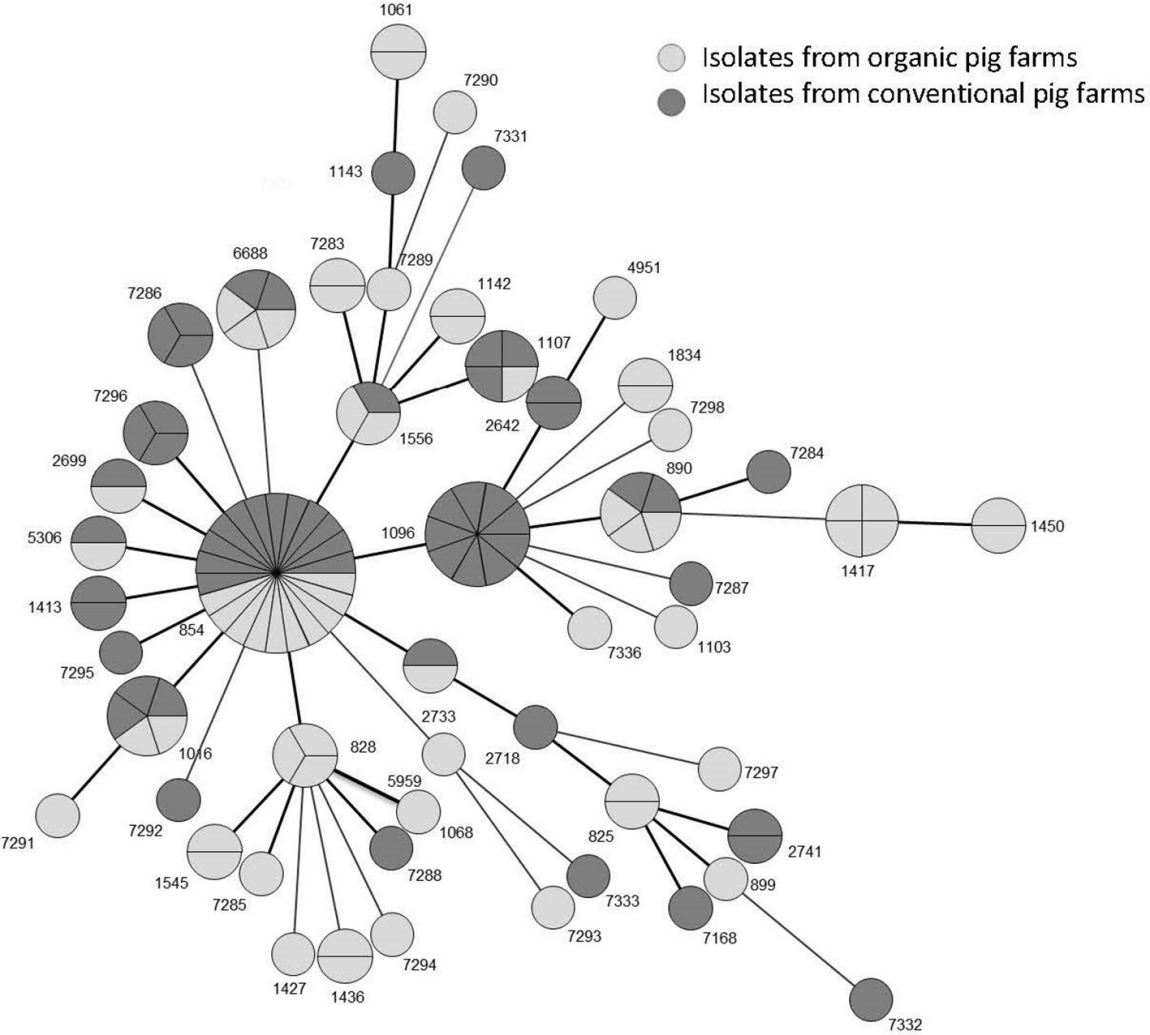
Distribution of the 120 isolates according their sequence type (ST) in a phylogenetic tree drawn using BioNumerics software.

Almost all isolates (91%) were from the ST828 CC; the other isolates (11) had no identified CC. Isolates were distributed among 50 STs (Figures 1, 2). The 62 isolates from organic farms were distributed into 34 STs and the 58 isolates from conventional farms into 26 STs. Nine STs (9/50), representing 41.6% of the isolates (50/120), were common to both organic and conventional farms, with a ST854 dominating (18.3% of the isolates, with 10 isolates from 8 organic farms and 12 isolates from 12 conventional farms). Among the 50 STs, 25 STs were only found in organic farm isolates (38 isolates) and 16 in conventional farm isolates (32 isolates), where ST1096 was found in 9 isolates. The diversity of the *Campylobacter* populations was higher in organic production systems with an index of diversity *D* = 0.96 _CI95%_[0.94–0.99] than in conventional production (*D* = 0.93 _CI95%_[0.89–0.97]). The distribution of the STs in the two production systems was not significantly different (χ^2^, *p* = 0.496).

The distribution of the STs was significantly different from the distribution of PFGE types (*p* = 0.003), indicating that STs are only weakly related to PFGE types.

Genetic diversity at individual MLST loci is shown in Table 3. The mean genetic diversity was higher in organic farms (0.435 ± 0.154) than in conventional farms (0.333 ± 0.206). Allelic diversity was higher for the *pgm* and *tkt* loci in both systems with a greater number of alleles for these two genes (Table 3). Between the two production systems, we observed a significant difference in genetic diversity for the *glnA, gltA*, and *uncA* locus, with higher genetic diversity for the organic system.

**Table 3:** Genetic diversity at individual loci of the multilocus sequence type for the 120 *C. coli* isolates from organic and conventional pig farms.

**Locus**	**Organic**			**Conventional**		
	**No. of allele**	**D**	**CI 95%**	**No. of allele**	**D**	**CI 95%**
*aspA*	4	0.211	0.08–0.35	4	0.134	0.01–0.25
*glnA*	4	0.544	0.48–0.61	3	0.220	0.08–0.35
*gltA*	4	0.472	0.35–0.60	3	0.133	0.01–0.25
*glyA*	5	0.240	0.10–0.38	7	0.391	0.23–0.55
*pgm*	8	0.439	0.29–0.59	8	0.492	0.34–0.65
*tkt*	8	0.617	0.51–0.72	10	0.693	0.60–0.79
*uncA*	5	0.527	0.44–0.62	2	0.267	0.14–0.40
Mean D		0.435 ± 0.154			0.333 ± 0.206	

*D, Simpson's index with 95% of confidence interval (CI 95%)*.

### Association between genetic profiles and antibiotic resistance profiles

The 120 *Campylobacter coli* were previously tested for their resistance to antibiotics. Resistant to tetracycline and erythromycin, and the number of resistances were significantly higher in isolates from conventional farms (Kempf et al., 2017).

The distribution of the ATB profiles was significantly different from the distribution of the PFGE types (*p* = 0.009) or the distribution of the STs (*p* = 0.012), indicating that ATB profiles are weakly related to PFGE types or STs.

However, the ST854 *C. coli* isolates predominantly showed resistance to tetracycline (90.9% of the ST854 isolates), streptomycin (90.9%), and susceptibility to erythromycin (86.3%).

Moreover, we noted that 76% of the isolates resistant to tetracycline were isolates with the allele 38 of the *glnA* gene (χ^2^, *p* = 0.007).

### Presence of the virulence genes

Regardless of pig origin, all the isolates carried the *ceuE, iam, ciaB*, and *flaA* genes. Moreover, all isolates from organic pigs carried also the *cadF* gene and the three *cdt* genes. *One* conventional isolate did not have the *cadF* gene and three conventional isolates lacked the three CDT genes. The plasmid gene *virB11* was detected only in eight isolates (7%), all from organic pigs. The presence of the plasmid was neither associated with a particular PFGE profile (see ^*^ in Figure 1) nor with a particular ST [ST 7285, 1450, 828 (2 isolates), 729, 1061, 825, and 724].

### Capacity of the isolates to adhere and invade Caco-2 cells

**Figure 3:**
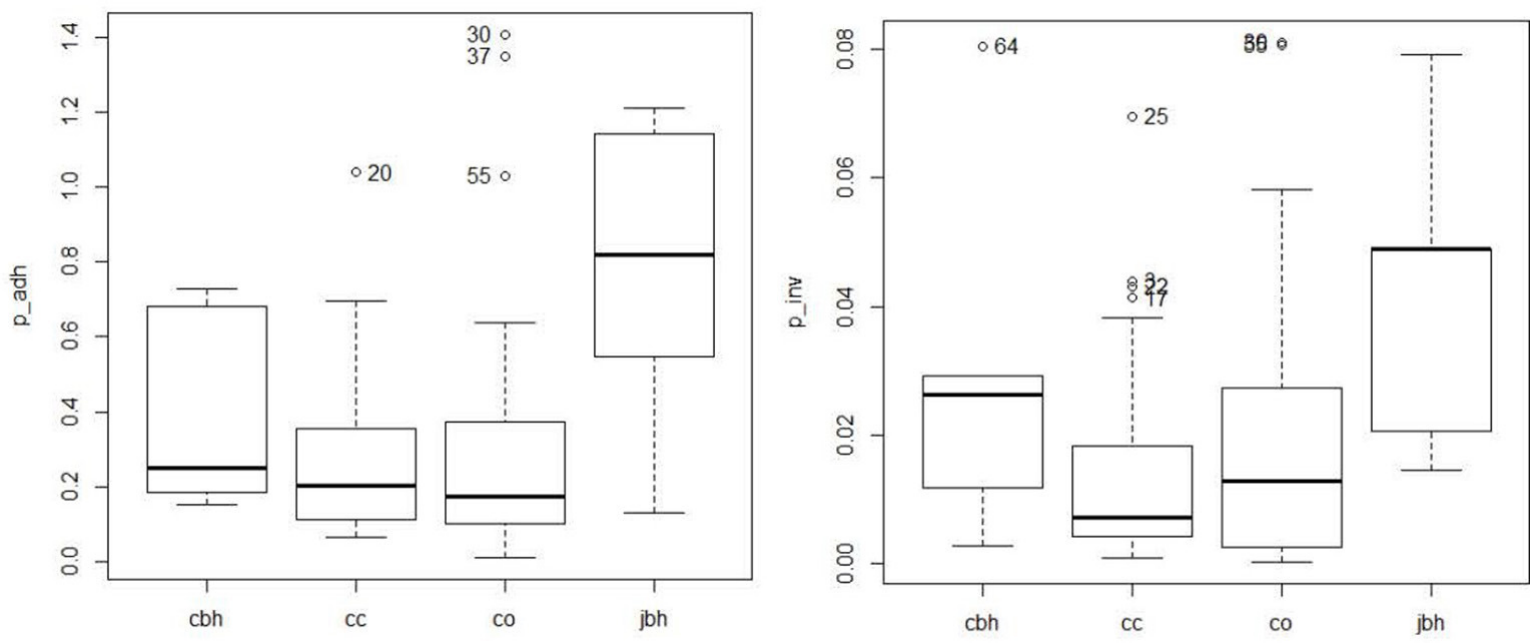
Distribution of the percentage of adhesion (p_adh) and the percentage of invasion (p_inv) according the origin of the isolates. cc, *C. coli* from conventional pig farm; co, *C. coli* from organic pig farm; cbh, *C. coli* from poultry and humans; jbk, *C. jejuni* from poultry and humans.

The percentage of adhesion and invasion of the 61 *C. coli* are indicated on the dendrogram in Figure 1. Adhesion was 0.30 and 0.28% on average for organic pig and conventional pig isolates, respectively, and invasion was 0.019 and 0.015% on average for organic pig and conventional pig isolates, respectively. There were no significant differences between the isolates from organic pigs and isolates from conventional pigs for adhesion (Mann-Whitney test, *p* = 0.523) or invasion (Mann-Whitney test, *p* = 0.590) (Figure 3).

The *C. coli* isolates' capacity to adhere and invade *Caco-2* cells was significantly lower than *C. jejuni* isolates isolated from poultry or humans (Mann-Whitney test, *p*-value for p_adh = 0.018 and *p*-value for p_inv = 0.014), but not significantly different from those of *C. coli* isolates isolated from poultry or humans (*p* > to 0.05) (Figure 3).

Among these isolates, eight had the plasmid gene *virB11*. There was no significant difference for adhesion between isolates with or without this gene (Mann-Whitney test, *p* = 0.058), but a significant difference was observed for invasion. Isolates with the *virB11* gene had a higher capacity to invade *Caco-2* cells than isolates without this gene (Mann-Whitney test, *p* = 0.040). We also observed that the human *C. jejuni* strain 81–176 carrying the *virB11* gene had a higher invasion capacity (0.079%) than the human *C. jejuni* strain NCTC 11168 without the *virB11* gene (0.015%).

**Figure 4:**
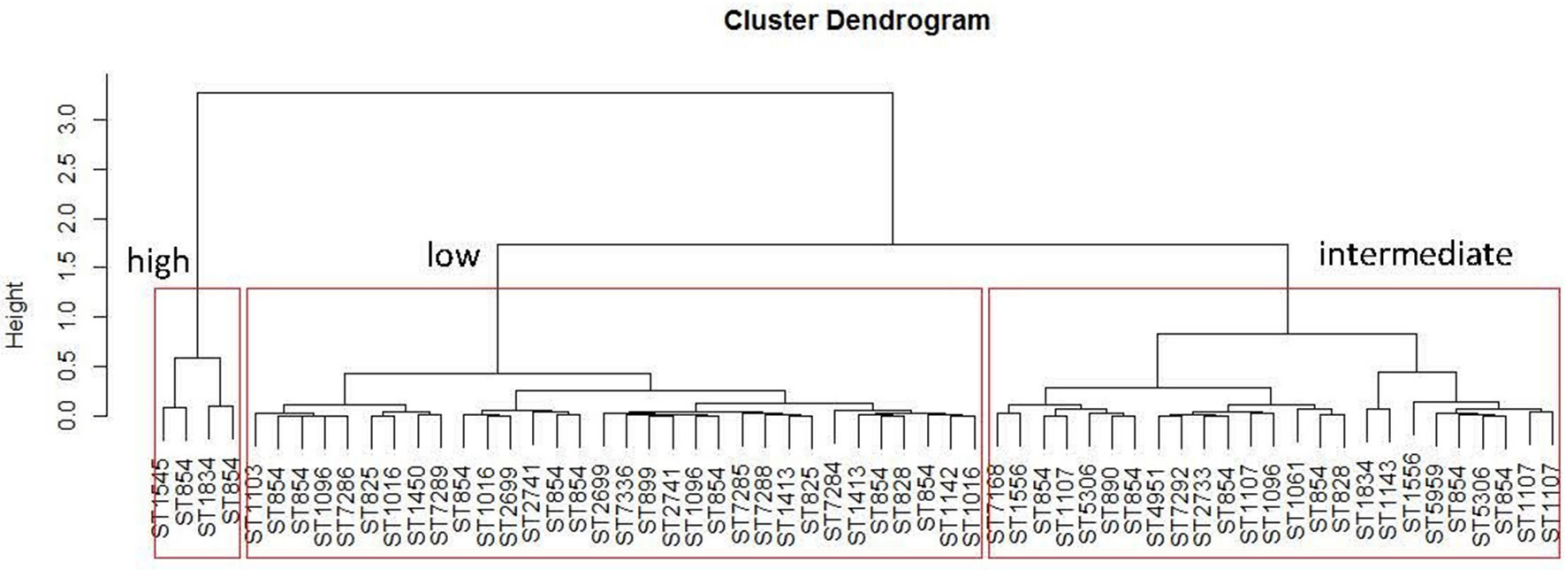
Distribution of the sequence types of the 61 *C. coli* isolates tested on *Caco2*-cells according their percentages of adhesion and invasion. The hierarchical clustering was done with R software using the method “hclust ward D2”. Three clusters were obtained for low, intermediate and high level pathogenicity for the *C. coli* tested.

The 61 *C. coli* were classified into three classes of pathogenicity (low, intermediate and high) from the hierarchical clustering done on the adhesion and invasion values (Figure 4). It was difficult to associate a ST with a virulence profile because there were too few isolates representing each ST, with the exception of ST854 for which 16 isolates were tested on *Caco-2* cells. These ST854 isolates were distributed among the three classes of pathogenicity.

## Discussion

In this study, the *C. coli* previously isolated from organic and conventional pig farms to test their resistance to antibiotics (Kempf et al., 2017) were typed by PFGE and MLST and tested for their virulence. We wanted to determine if the production system has an impact on the genetic diversity and on the virulence of these isolates excreted by the pigs.

First, our study focused only on *C. coli*, the only species that was isolated from our pigs. We expected that outdoor pigs would be more exposed to *C. jejuni* from the environment because wild animals, particularly birds, can shed *Campylobacter* species other than *C. coli* (Greig et al., 2015). However, although Jensen et al. (2006) showed that the paddock environment of organic pigs was contaminated by non-pig strains, e.g., by wild fauna strains, they did not demonstrate that these strains could contaminate pigs, at least not *C. jejuni* strains. Pigs show a dominance of *C. coli* (Nielsen et al., 1997; Alter et al., 2005; Boes et al., 2005) and *C. jejuni* may co-exist with *C. coli* in pigs, but *C. jejuni* is typically present at in numbers that are 10–100-fold lower than *C. coli* (Madden et al., 2000; Jensen et al., 2005). In our study, we tested only two to three isolates per farm which may explain also why we never detected *C. jejuni*.

PFGE and MLST revealed high genetic diversity in our *C. coli* populations, as previously observed in French pig farms using PFGE (Denis et al., 2011). Some of the STs have been already described in other countries: ST854 and ST2718 from pig livers in Germany (von Altrock et al., 2013), ST854, ST890, ST1068, ST1096, ST1142, ST1413, ST1417, ST1436, and ST1450 from pig farms in USA (Thakur et al., 2006), and ST828, ST854, ST890, ST1016, ST1061, ST1096, ST1413, ST1556, ST2733, and ST4951 from pig feces at slaughterhouses in Switzerland (Egger et al., 2012). The most prevalent ST in our study was ST854. This ST was also reported to be predominant in pig farms in the USA (Quintana-Hayashi and Thakur, 2012) and has been detected all along the production chain, from pig farms to retail pork meat products (Abley et al., 2012). It is also the most frequently recovered ST from surface of pig livers in Germany (von Altrock et al., 2013). In our study, the ST854 isolates predominantly exhibited the TET+STR+ERY− phenotype whereas the CIP/NAL+TET+ phenotype was mainly found for this ST in the USA study (Quintana-Hayashi and Thakur, 2012).

We also noted that allelic diversity was higher at the *pgm* and *tkt* loci than the other virulence loci, with a greater number of alleles for these two loci. Quintana-Hayashi and Thakur (2012) also observed higher allelic diversity for the *tkt* and *glyA* loci in *C. coli* populations from swine farms.

Although the genetic diversity was higher in organic production than in conventional production for both typing methods, PFGE and MLST, there were no significant differences, except at the locus level for the *glnA, gltA*, and *uncA* genes. There were a higher number of alleles for these genes when isolates were from organic pigs. Finally, we were not able to clearly demonstrate that organic production practices with little or no use of antibiotics and outdoor access for pigs promotes a higher number of *Campylobacter* genotypes. There were common *Campylobacter* genotypes shared between both production systems. We already identified these PFGE types on French pig production farms in 2008 (Denis et al., 2011) and two STs (ST584, ST890) were also isolated by Thakur et al. (2006) from conventional and antimicrobial-free pig farms in the USA. Our results suggest that these common genotypes are adapted to the pig and that other genotypes are likely specific to the farm environment where the pigs are grown.

The production system does not select for specific virulence gene profiles, with the exception of the plasmid gene *virB11*. Almost all the isolates carried the *ceuE, iam, ciaB, flaA, cadF* genes, and the three *cdt* genes. High prevalence for these genes has been obtained on *C. coli* in many studies (Bang et al., 2003; Rozynek et al., 2005; Wieczorek and Osek, 2010, 2013; Andrzejewska et al., 2011; Acik et al., 2013; Khoshbakht et al., 2013) except for the *ciaB* gene. The detection of *ciaB* varies with species and study, ranging from 20% for *C. coli* (Wieczorek and Osek, 2010; Acik et al., 2013) up to 100% for *C. jejuni* (Datta et al., 2003; Feodoroff et al., 2010). This discrepancy may be due to the primers used; we designed new primers that facilitated the detection of the *ciaB* gene in all our *C. coli* isolates.

The plasmid gene *virB11*was detected in eight isolates, all from organic pigs. This gene has also been detected in 28% of the *C. jejuni* strains isolated from free-range broiler flocks (Hanning et al., 2010) and with a low prevalence for *C. coli* from various origins (Wieczorek and Osek, 2010, 2013; Acik et al., 2013). The low frequency of these isolates did not allow us to conclude that their presence in organic farms is related to the management of this type of production system.

There was no difference in the pathogenicity between organic and conventional *C. coli* pig isolates when tested on *Caco*-2 human intestinal cells. Moreover, different levels of pathogenicity were observed for the ST854 isolates, the most prevalent ST, regardless of the production system. Our *C. coli* isolates have low adhesion and invasion capacities, similar to *C. coli* from poultry and humans, compared with *C. jejuni* strains from poultry and humans. Guyard-Nicodème et al. (2013) reported similar results between the two species in a comparison of *C. jejuni* and *C. coli* strains isolated from poultry.

We showed that the isolates with the plasmid gene *virB11* had a higher invasion capacity than isolates without this gene. Moreover, the human *C. jejuni* 81–176 strain carrying the plasmid also showed higher invasion capacity than the human *C. jejuni* NCTC 11168, which does not possess the vi*rB11* gene. This relationship between high invasion and presence of the plasmid gene *virB11* has previously been reported (see Bacon et al., 2002).

## Conclusion

Our study could not conclusively demonstrate that the type of pig production system influences the *C. coli* population. We confirmed the high genetic diversity of *C. coli* in pigs in France, and showed that isolates sharing the same ST may show different levels of pathogenicity. This study helped improve the detection of virulence genes in *C. coli*, a species less studied than *C. jejuni*, and provided data on the virulence of this species, and more particularly of *C. coli* isolated from pigs.

## Author contributions

MD: Conception of the study, analysis and interpretation of data, and drafting of the manuscript; BN, VR, KB, MC, AK: Acquisition of the isolates and data; MD, AK: Critical revision of important intellectual content; All authors: Final approval of the version to be published and accountable for all aspects of the work, ensuring that questions related to the accuracy or integrity of any part of the work were appropriately investigated and resolved.

### Conflict of interest statement

The authors declare that the research was conducted in the absence of any commercial or financial relationships that could be construed as a potential conflict of interest.
